# The impact of multi-target stool DNA testing in clinical practice in the United States: A real-world evidence retrospective study

**DOI:** 10.1016/j.pmedr.2022.102045

**Published:** 2022-11-07

**Authors:** Lesley-Ann Miller-Wilson, Paul Limburg, Leah Helmueller, Maria João Janeiro, Paul Hartlaub

**Affiliations:** a Exact Sciences Corporation, Madison, WI, USA; b Mayo Clinic, Rochester, MN, USA; c Maple Health Group, NY, USA; d Ascension Medical Group, Brown Deer, WI, USA

**Keywords:** Colorectal Cancer, Screening, Real-World, Colonoscopy Stool-based tests

## Abstract

Widely endorsed screening modalities for colorectal cancer (CRC) include structural visualization (e.g. colonoscopy) and stool-based tests including multitarget stool DNA (mt-sDNA), fecal immunochemical tests (FIT), or high-sensitivity guaiac-based fecal occult blood tests (gFOBT). However, CRC screenings are underutilized, hence understanding the screening utilization trends is important, particularly with respect to the newest guideline-endorsed option (mt-sDNA). The objective of this study was to assess patterns in overall CRC screenings following clinical availability of the mt-sDNA test among average-risk individuals in the Ascension Wisconsin healthcare system focusing primarily on individuals aged 50–75 years old. We also reported CRC screening behaviors among individuals < 50 and > 75 years old. Electronic medical records of individuals aged ≥ 40 years from 2015 to 2018 were reviewed to identify average-risk and screen-eligible members. For those with screening data available, we determined the proportion who were up-to-date with any United States Preventive Services Task Force (USPSTF) recommended screening strategy; the number of screening tests performed in the measurement year; and the distribution of screening modalities. Temporal trends were assessed using regression analysis, including subgroup analyses across age groups and screening modalities. A total of 172,045 unique patients aged ≥ 40 years were included, of which 115,708 individuals aged 50–75 years. When considering all individuals up-to-date and screened in the measurement year, overall adherence increased significantly over the 4-year study period, from 39,105 to 49,698 patients or 47 % to 59 % (p < 0.0001). The screening incidence between 2015 and 2018 increased from 19.44 to 23.66 tests per 1,000 persons for gFOBT and FIT, a 1.2-fold increase, and from 6.54 to 29.78 tests per 1,000 persons for mt-sDNA (p < 0.05), a 4.6-fold increase. During the same time period, the screening incidence of colonoscopy decreased from 119.99 to 110.58 tests per 1,000 persons, corresponding to a decrease of 8 %. Similar patterns in screening incidence rates were observed among those aged < 50 and > 75 years old. Growing adoption, higher preference, and the broad availability of mt-sDNA testing may be associated with an increase in overall CRC screening rates in the average-risk population, in parallel with a slight increase in the use of other non-invasive CRC screening tests.

## Highlights

• CRC screening tests are underutilized among average-risk adults aged ≥40 years.
• CRC screening adherence increased from 35.6% to 46.6% from 2015 to 2018.
• Screening incidence decreased for colonoscopy and increased for mt-sDNA tests.
• Non-invasive at-home screening tests may offer a practical option for initial screening.

## 1 Introduction

Colorectal cancer (CRC) is the third most commonly diagnosed cancer and the second leading cause of cancer-related deaths among men and women combined in the United States (US). The American Cancer Society (ACS) estimated around 150,000 new cases of CRC and over 53,000 projected deaths from CRC in 2021 (American Cancer Society, 2021). In general, CRC is more prevalent among persons aged 65–74 years (American Cancer Society, 2021). Nonetheless, the CRC incidence rates in persons aged 40 to 49 years have increased by almost 15 % from 2000 to 2002 to 2014–2016 (Montminy et al., 2021 Feb).

While research has shown that early detection of CRC through regular screenings improves CRC-related outcomes and reduces mortality, the reported CRC screening rate of 68.8 % in 2018 is considerably lower than the targeted 80 % screening participation goal (National Health Interview Survey Public Use Data File, 2019, National Colorectal Cancer Roundtable, 2019, Joseph et al., 2018). The suboptimal CRC screening rate persists, despite the availability of multiple recommended test options. Most current average-risk CRC screening guidelines recommend that adults aged >= 45 years initiate screening with an endoscopic, radiologic, or stool-based test, with final selection based on factors such as test availability and individual preference (Wolf et al., 2018, US Preventive Services Task Force et al., 2021). Recognizing the importance of increasing CRC screening engagement, the United States Preventive Services Task Force (USPSTF) and other major guideline review groups, including the ACS and National Comprehensive Cancer Network, recommend multiple CRC screening strategies with equal positioning. The USPSTF guidelines recommend any of the following options: gFOBT, annually; FIT, annually; multi-target stool DNA (mt-sDNA) test, every 1 to 3 years; colonoscopy, every 10 years; CT colonography, every 5 years; flexible sigmoidoscopy, every 5 years; or flexible sigmoidoscopy every 10 years with annual FIT testing (US Preventive Services Task Force et al., 2021). Further, all the positive noninvasive test results should be followed by a timely colonoscopy for diagnostic purposes as delays in follow-up colonoscopy after positive results on stool-based tests are associated with increased risks for adverse CRC outcomes, including death (US Preventive Services Task Force et al., 2021, Corley et al., 2017, Doubeni et al., 2019, Azulay et al., 2021).

The mt-sDNA test is the most recently endorsed option for average-risk CRC screening, being added to the USPSTF recommendations in 2016. Mt-sDNA testing was designed for the qualitative detection of colorectal neoplasia-associated DNA markers and the presence of occult hemoglobin in human stool. In 2019, mt-sDNA was approved for CRC screening in average-risk adults beginning at age 45 years, rather than age 50 years. The mt-sDNA test analyzes patients’ stool for the presence of 11 molecular markers, which may indicate the presence of CRC or advanced adenoma. Based on combined results of the DNA markers and hemoglobin, a qualitative “positive” or “negative” test result is provided. Patients with a positive result should be referred for a follow-up colonoscopy, and patients with a negative result should continue with a regular CRC screening schedule.

The clinical performance of the mt-sDNA test compared to FIT tests was demonstrated in a large, cross-sectional clinical study (Imperiale et al., 2014). Among the enrolled 9,989 asymptomatic participants at average risk for CRC, mt-sDNA testing detected significantly more cancers than FIT but also had more false-positive results. As compared to colonoscopy and other structural screening tests, the stool-based tests offer advantages including the non-invasive nature of the tests, no bowel preparation, no changes in medications or diet (except for FOBT), and the convenience of performing the test at home avoiding the travel/preparation time. Due to these benefits, individuals at average risk for CRC may prefer stool-based screening tests over more invasive options (Pickhardt et al., 2021). A recent national survey by Zhu et al found that when survey respondents were presented with a choice, the majority of the survey respondents preferred stool-based tests (mt-sDNA and FIT/gFOBT) over colonoscopy. When given a choice between mt-sDNA and FIT/gFOBT), as much as 66.9 % of the respondents preferred mt-sDNA (Zhu et al., 2021).

Although recent studies show a favorable trend toward mt-sDNA usage, the impact of mt-sDNA adoption in the real-world setting has not been fully explored. The objective of this retrospective medical record review was to determine the impact of mt-sDNA test adoption on CRC screening adherence, utilization, and proportional mix in a real-world healthcare setting. We assessed CRC screening adherence calculated as a proportion of average-risk, screen-eligible individuals, active in the Ascension Wisconsin Healthcare System who are up to date with CRC screening by any USPSTF recommended screening strategy within each measurement year. We further calculated CRC screening test mix as utilization rates and screening incidence of mt-sDNA, FIT, gFOBT, screening colonoscopy, CT colonography (CTC), and sigmoidoscopy (SIG), and other USPSTF recommended CRC screening options offered by the system within each measurement year for individuals who were active in the Ascension Wisconsin Healthcare System during the preceding 3 years.

## 2 Methods

### 2.1 Study design and data sources

We conducted a retrospective electronic medical record review including average risk, CRC screening-eligible individuals aged 40 years and older identified within Ascension Wisconsin healthcare system. At the time of the study, the USPSTF guidelines recommended starting CRC screening at age of 50 years. However, we wanted to assess CRC screening patterns among the younger population as well, hence the inclusion of individuals aged 40 years and above in the study. This was motivated by evidence that populations<50 years of age saw steeper increases in CRC incidence and mortality in recent years (American Cancer Society, 2020). It is also important to understand the risk among individuals younger than 50 years as these individuals carry a higher risk of CRC as they age. Based on the clinical and epidemiological evidence, the 2018 ACS guidelines recommended starting age of CRC screening to be<50 (Wolf et al., 2018). Note that during the study time period, the clinical guidelines recommended CRC screening for individuals aged 50 years and older (primarily those aged between 50 and 75 years). Hence, we evaluated study outcomes separately for the 50–75-year-old population and those<50 and above 75 years to understand utilization patterns in these age groups.

The study measurement periods were defined as January 1 to December 31 of 2015, 2016, 2017, and 2018. The study was approved by the Vanderbilt Institutional Review Board (IRB# 200285). All data were de-identified in the extraction phase prior to being transferred as per the data transfer agreement.

### 2.2 Study population

**Fig. 1:**
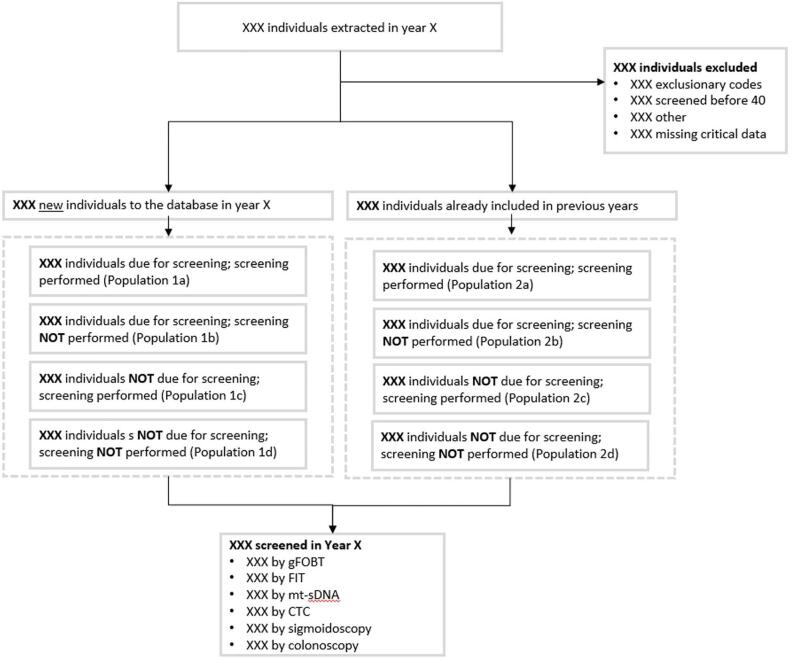
Study Flow Schema.

The study population included individuals aged 40 years and older at the time of CRC screening test results date (procedure date for screening colonoscopy). Patients were required to have at least 1 visit within the previous 36 months to a provider from the Ascension Wisconsin healthcare system in Family Medicine, Internal Medicine, or Geriatrics practices. The primary analysis included individuals aged 50–75 years and had a CRC test date recorded in the data. For some women, a visit to the gynecologist may be considered a well-woman visit. As a result, cancer screening (including CRC) may be discussed. However, OB/GYNs often focus more as specialty care providers, so we excluded women with anOB-GYN visit for this analysis. Evidence of above-average or high-risk history prior to screening was determined by the presence of at least one International Classification of Diseases (ICD)-9/ICD-10 code indicating the presence, history, or symptoms of - Benign or malignant colorectal neoplasms, colorectal polyps, inflammatory bowel disease (ulcerative colitis or Crohn’s disease), family history of CRC, familial adenomatous polyposis, or hereditary nonpolyposis CRC (Appendix 1). The study flow schema is presented in Fig. 1.

### 2.3 Study measures

Member demographic variables included age (calculated at the end of the measurement year on Dec 31st), gender (Male, Female), race (American Indian/Pacific Islander, Asian, Black, White, Other); Hispanic (Yes, No), payor type (Commercial, Medicare, Medicaid, Self Pay, Other, Unknown). Please note that the race categories including “Other” are reported as available in the health system dataset. Unfortunately, with the data extract that we have access to from the health system, it is not possible to break down these categories further. Study outcomes included a proportion of individuals (for whom screening data were available) who were up to date with a USPSTF recommended screening strategy (screening adherence) calculated among the overall population as well as for those who were due for CRC screening; the number of screening tests performed in the measurement year (screening incidence); and distribution of screening modalities (test mix). All study outcomes were assessed for January 1 to December 31 of 2015, 2016, 2017, and 2018 for the overall population and by age groups.

### 2.4 Statistical analyses

Descriptive statistics including means (standard deviations) for continuous variables and frequencies for categorical variables were calculated. Binomial regressions were used to assess the trends in screening adherence over time (2015–2018). Gaussian regressions were used to assess the trends in screening incidences over time (2015–2018) for the overall cohort and across screening modalities. All analyses were performed using R Statistical Software (Vienna, Austria).

## 3 Results

A total of 115,708 unique patients aged above 50 years were included. Across all 4 years (2015–2018), around 53 % members were females, over 80 % whites, and the majority were non-Hispanics (Table 2).

**Table 1:** Study Sample.

	Base case (without OBGYN only)							
**Number of unique patients**	**01/01/2015–12/31/2015**		**01/01/2016–12/31/2016**		**01/01/2017–12/31/2017**		**01/01/2018–12/31/2018**	
**Total extracted**	143,354		146,291		145,939		143,896	
Excluded due to exclusionary ICD-9 codes	5,519	3.8 %	5,973	4.1 %	6,282	4.3 %	6,774	4.7 %
Excluded due to screening before age of 40	0	0.0 %	0	0.0 %	0	0.0 %	0	0.0 %
Excluded due to missing critical data (i.e. test dates)*	0	0.0 %	0	0.0 %	0	0.0 %	0	0.0 %
Excluded due to other**	10,712	7.5 %	10,204	7.0 %	9,872	6.8 %	9,332	6.5 %
Total excluded	16,006	11.2 %	15,950	10.9 %	15,926	10.9 %	15,870	11.0 %
**Total Remaining**	**127,348**	**88.8 %**	**130,341**	**89.1 %**	**130,013**	**89.1 %**	**128,026**	**89.0 %**
**New patient**	11,646	9.1 %	10,447	8.0 %	7,650	5.9 %	6,768	5.3 %
due for screening this year and performed it	1,165	10.0 %	1,280	12.3 %	925	12.1 %	849	12.5 %
due for screening this year and did not perform it	9,772	83.9 %	8,419	80.6 %	6,367	83.2 %	5,709	84.4 %
NOT due for screening this year and performed it	18	0.2 %	36	0.3 %	7	0.1 %	6	0.1 %
NOT due for screening this year and did not perform it	691	5.9 %	712	6.8 %	351	4.6 %	204	3.0 %
**Existing patient**	115,702	90.9 %	119,894	92.0 %	122,363	94.1 %	121,258	94.7 %
due for screening this year and performed it	8,101	7.0 %	8,308	6.9 %	7,959	6.5 %	7,029	5.8 %
due for screening this year and did not perform it	72,278	62.5 %	69,726	58.2 %	66,865	54.6 %	62,724	51.7 %
NOT due for screening this year and performed it	2,903	2.5 %	4,382	3.7 %	5,043	4.1 %	4,891	4.0 %
NOT due for screening this year and did not perform it	32,420	28.0 %	37,478	31.3 %	42,496	34.7 %	46,614	38.4 %

	Base case (without OBGYN only)							
Number of unique patients	01/01/2015–12/31/2015		01/01/2016–12/31/2016		01/01/2017–12/31/2017		01/01/2018–12/31/2018	
**CRC Screening by modalities**								
Screened this year by any modality	12,187	9.6%	14,006	10.7%	13,934	10.7%	12,775	10.0%
Screened this year by gFOBT/FIT	2,416	19.8%	3,091	22.1%	2,683	19.3%	2,157	16.9%
Screened this year by mt-sDNA	400	3.3%	686	4.9%	953	6.8%	1,404	11.0%
Screened this year by CTC	9	0.1%	15	0.1%	13	0.1%	1	0.0%
Screened this year by SIG	13	0.1%	45	0.3%	53	0.4%	57	0.4%
Screened this year by colonoscopy	9,349	76.7%	10,169	72.6%	10,232	73.4%	9,156	71.7%

Notes: * *assumed, since blank cells could correspond to tests not performed but also to missing data.

**NA for sex and OB/GYN visits only.

**Table 2:** Demographic characteristics (aged 50–75 years).

	2015		2016		2017		2018	
	N	%	N	%	N	%	N	%
**Total**	82,553	100 %	84,952	100 %	84,941	100 %	83,721	100 %
**Sex**								
Males	38,276	46.37 %	39,844	46.90 %	40,027	47.12 %	39,420	47.08 %
Females	44,277	53.63 %	45,108	53.10 %	44,914	52.88 %	44,301	52.92 %
**Race**								
American Indian/Pacific Islander	206	0.25 %	229	0.27 %	232	0.27 %	236	0.28 %
Asian	1,035	1.25 %	1,165	1.37 %	1,241	1.46 %	1,333	1.59 %
Black	9,970	12.08 %	10,588	12.46 %	10,680	12.57 %	10,363	12.38 %
White	68,516	83.00 %	70,038	82.44 %	69,868	82.25 %	69,049	82.48 %
Other	2,826	3.42 %	2,932	3.45 %	2,920	3.44 %	2,740	3.27 %
**Hispanic**								
Yes	3,999	4.84 %	4,400	5.18 %	4,676	5.50 %	4,854	5.80 %
No	78,554	95.16 %	80,552	94.82 %	80,265	94.50 %	78,867	94.20 %
**Payment type**								
Commercial	8,605	10.42 %	9,804	11.54 %	10,764	12.67 %	11,408	13.63 %
Medicare	15,371	18.62 %	15,182	17.87 %	14,447	17.01 %	13,314	15.90 %
Medicaid	1,002	1.21 %	1,147	1.35 %	1,218	1.43 %	1,170	1.40 %
Self Pay	2,997	3.63 %	3,472	4.09 %	3,675	4.33 %	3,417	4.08 %
Other	48,415	58.65 %	52,164	61.40 %	54,367	64.01 %	54,498	65.09 %
Unknown	6,163	7.47 %	3,183	3.75 %	470	0.55 %	4	0.00 %

### 3.1 Screening adherence

**Fig. 2:**
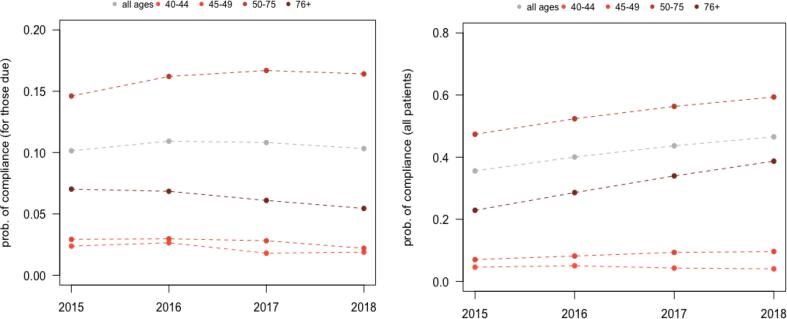
Screening Adherence (Overall and by Age groups).

When considering individuals up-to-date and screened in the measurement year, overall adherence among those aged 50–75 year old increased significantly over the 4-year study period. For from 47.4 % to 59.4 % (p < 0.0001) and (Fig. 2). However, the trends were different for the population due for CRC screening. For individuals aged 50–75 years due for CRC screening, adherence rates increased from 14.6 % in 2015 to 16.4 % in 2018 (p < 0.0001).

### 3.2 CRC-screening test-mix

#### 3.2.1 Year-over-year screening proportions:

The CRC screening proportions from 2015 to 2018 are presented in Table 3. Overall, among those who were up-to-date with their CRC screening, the proportion of members receiving mt-sDNA increased significantly from 3.5 % in 2015 to 12.0 % in 2018 (p < 0.0001). During the same period, the proportion of members aged 50–75 years old receiving screening colonoscopy decreased from 79.7 % in 2015 to 72.7 % in 2018.

**Table 3:** CRC Screening Test Mix – Proportion (aged 50–75 years).

	**01/01/2015–12/31/2015**	**01/01/2016–12/31/2016**	**01/01/2017–12/31/2017**	**01/01/2018–12/31/2018**	**Linear trend**	
					**p-value**	**direction**
**CRC Screening Test Type**						
High sensitivity gFOBT (gFOBT) / FIT	16.68 %	19.56 %	17.22 %	14.96 %	<0.0001	negative
mt-sDNA	3.52 %	5.42 %	7.45 %	12.04 %	<0.0001	positive
CT colonography	0.07 %	0.06 %	0.07 %	0.01 %	<0.1	negative
Screening Colonoscopy	79.67 %	74.72 %	75.01 %	72.68 %	<0.0001	negative
Sigmoidoscopy	0.07 %	0.24 %	0.26 %	0.31 %	<0.001	positive

#### 3.2.2 Year-over-year screening incidence and rates:

Overall, screening incidence between 2015 and 2018 increased significantly for mt-sDNA (Table 4). screening incidence between 2015 and 2018 increased from 19.44 to 23.66 tests per 1,000 persons for gFOBT and FIT, a 1.2-fold increase, and from 6.54 to 29.78 tests per 1,000 persons for mt-sDNA (p < 0.05), a 4.6-fold increase. During the same time period, the screening incidence of colonoscopy for this age group decreased from 119.99 to 110.58 tests per 1,000 persons, corresponding to a decrease of 8 %.

**Table 4:** CRC Screening Test Mix - Screening Incidence and Rates (aged 50–75 years).

	Screening incidence for those due for CRC screening, per 1000						Screening incidence for all, per 1000					
	01/01/2015–12/31/2015	01/01/2016–12/31/2016	01/01/2017–12/31/2017	01/01/2018–12/31/2018	Linear trend		01/01/2015–12/31/2015	01/01/2016–12/31/2016	01/01/2017–12/31/2017	01/01/2018–12/31/2018	Linear trend	
CRC Screening Test Type					p-value	direction					p-value	direction
**Screening Incidence**												
Any screening modality	146.09	162.03	166.90	164.12	NS	N/A	120.52	136.68	138.07	128.99	NS	N/A
High sensitivity gFOBT (gFOBT)	16.45	20.77	15.86	7.71	NS	N/A	15.95	19.94	14.98	8.97	NS	N/A
FIT	2.99	6.48	10.36	15.94	<0.01	positive	4.14	6.79	8.79	10.33	<0.01	positive
Mt-sDNA (COLOGUARD)	6.54	11.80	17.93	29.78	<0.05	positive	4.24	7.40	10.29	15.53	<0.05	positive
CT colonography (CTC)	0.08	0.04	0.00	0.00	<0.1	negative	0.08	0.08	0.09	0.01	NS	N/A
Screening Colonoscopy (SC)	119.99	122.83	122.62	110.58	NS	N/A	96.01	102.13	103.57	93.75	NS	N/A
Sigmoidoscopy (SIG)	0.04	0.10	0.13	0.10	NS	N/A	0.08	0.33	0.35	0.39	NS	N/A
**Screening Rates**												
Any screening modality	120.52	136.68	138.07	128.99	NS	N/A	473.70	523.71	563.37	593.61	<0.01	positive
High sensitivity gFOBT (gFOBT)	15.95	19.94	14.98	8.97	NS	N/A	22.49	29.18	27.62	23.72	NS	N/A
FIT	4.14	6.79	8.79	10.33	<0.01	positive	4.19	8.06	11.48	14.79	<0.001	positive
Mt-sDNA (COLOGUARD)	4.24	7.40	10.29	15.53	<0.05	positive	4.26	10.81	19.58	30.58	<0.01	positive
CT colonography (CTC)	0.08	0.08	0.09	0.01	NS	N/A	0.12	0.19	0.28	0.24	NS	N/A
Screening Colonoscopy (SC)	96.01	102.13	103.57	93.75	NS	N/A	442.32	474.89	503.63	523.29	<0.01	positive
Sigmoidoscopy (SIG)	0.08	0.33	0.35	0.39	NS	N/A	0.30	0.58	0.78	1.00	<0.01	positive

#### 3.2.3 CRC screening utilization among younger (<50 year old) and older (75+) populations

Overall demographic characteristics are presented in Appendix Table 1. Similar to the 50–75 cohort, CRC screening adherence increased significantly from 2015 to 2018 among younger population (11.6 % to 13.7 %; p < 0.0001), driven mostly by individuals aged 45 to 49 year old (7.0 % to 9.6 %; p < 0.0001). For this cohort, colonoscopy screening incidence decreased from 2015 to 2018 while other screening incidence rates remained unchanged (Appendix Table 2). Similarly, for individuals aged above 75 years who were up-to date and screened during measurement years, screening adherence increased from 22.9 % to 38.7 % (p < 0.0001). For those due for CRC screening, adherence rates decreased from 7.0 % in 2015 to 5.5 % in 2018 (p < 0.0001).

## 4 Discussion

This retrospective study examined real-world trends in CRC screening proportion and incidence rates from 2015 to 2018 using comprehensive medical records of members who visited the Ascension Wisconsin healthcare system, providing robust population-based estimates. Until 2018, clinical guidelines recommended CRC screenings for average-risk individuals aged 50 years and above. More recently, the CRC screening age was reduced to 45 years old. Since our study period ranges from 2015 to 2018, we assessed outcomes focusing primarily on members aged between 50 and 75 years. Further, we also evaluated outcomes in other age groups including 40–44 years, 45–49 years, and above 75 years. Additionally, the study offers a timely assessment of CRC screening proportions and incidence rates during four consecutive 12-monthly periods following the approval of the mt-sDNA test in the US in 2014.

In this study, adherence to CRC screening tests increased from 2015 (35.6 %) to 2018 (45.6 %) overall, and by age group. For the 50–75 age group, adherence to CRC screening tests increased from 47.4 % in 2015 to 59.4 % in 2018. A recent report using data from the Behavioral Risk Factor Surveillance System (BRFSS) survey showed that the overall proportion of U.S. adults ages 50–75 with “up-to-date” CRC screening increased from 65.5 % in 2012 to 67.3 % in 2016 (BRFSS Survey Data and Documentation, 2020). According to the National Health Interview Survey (NHIS) data, rates of up-to-date CRC screening steadily increased from approximately 35 % to 62 % between 2000 and 2015 (Hall et al., 2018). Our study included recent years (2015–2018) and showed similar trends indicating a steady increase in CRC screening tests from 2015 to 2018. However, the adherence rates across all years in the current study were lower than the previously reported rates. Note that the previous studies reporting trends in CRC adherence rates primarily used survey data. Differences in the adherence rates may be due to the underlying differences in the study population, data type, data availability, and definition of CRC adherence. Future studies should focus on understanding the impact of sociodemographic variables such as sex, race, and ethnicity on CRC screening uptake.

Among those who were screened for CRC, the annual CRC screening incidence rates were stable from 2015 to 2018, while CRC test-specific rates increased for mt-sDNA testing, the newest guideline-endorsed option for average-risk CRC screening and decreased for screening colonoscopy supporting the shift in the trend to non-colonoscopy screening options. These results are consistent with a study conducted by Rutten et al among 5,818 residents of Olmsted County, MN eligible and due for CRC screening (Finney Rutten et al., 2020). Similar to the current study, the Rutten study reported a significant reduction in the incidence of screening colonoscopy 66.6 to 52.5 per 1000 eligible population between 2016 and 2018 (p < 0.0001). During the same period, mt-sDNA screening incidence increased significantly from 38.2 to 57.7 per 1000 eligible population (p < 0.0001). Note that the prior study was limited to data from one county in MN. The current study adds to the literature by including robust population estimates overall and by age groups obtained from a larger clinical database. Other real-world studies have also reported increased utilization of mt-sDNA tests since its availability in the US starting in 2015 (Finney Rutten et al., 2020, Fisher et al., 2021, Limburg et al., 2021).

These trends may suggest a growing interest among average-risk adults and/or clinicians in non-invasive stool-based screening that can be performed at home (Zhu et al., 2021). Several barriers to colonoscopy have been identified in previous studies including bowel preparation, requiring dietary changes, travel time, anxiety around invasive testing, and concerns related to the procedure-related complications. In contrast, distinct advantages of stool-based tests include the convenience of performing tests at home, non-invasive nature, and no prior preparation. In addition, the mt-sDNA test is supported by a robust patient navigation program, as well as 24/7 telephonic assistance available for those who may need it offering a helpful tool to clinicians and patients who want to seek additional information regarding CRC screening procedures (Weiser et al., 2021). It is also important to note that the USPSTF support for CRC screening at age 45 followed the recommendation from the ACS guidelines in 2018. As the current study population led up to 2018, those who elected screening before age 50 may represent an average-risk (based on criteria described under the “Study Population” section above) yet highly motivated group. For this group, CRC screening would likely not have been covered by insurance resulting in a patient expense, which may have resulted in individuals choosing a cheaper, noninvasive test rather than colonoscopy. The increase in mt-sDNA uptake is not surprising since mt-sDNA was a relatively new test to the market. However, we found that during the study years, utilization of SBTs (FIT and mt-sDNA combined) increased significantly primarily driven by higher utilization of mt-sDNA.

Several study limitations are worth noting. First, there are limitations associated with using administrative data. There may be potential coding errors leading to selection bias. However, due to the mandated coverage of CRC screenings under the Affordable Care Act in 2011, the use of ICD codes would likely have a low error rate during our study time frame. Individuals receiving screening outside of the participating organization were not included, thus the study has limited generalizability beyond members outside the Ascension health system. Further, we did not have information on when subjects became up-to-date prior to their enrollment, thus they may have been due for screening during the study period. However, we did not capture this, possibly leading to an overestimation of CRC screening modality proportions and screening incidence rates. Another limitation is a lack of extensive socioeconomic data for the study population. Although we had data on insurance status, more than half of the population was categorized as having Other/Unknown insurance limiting the interpretation of the findings, particularly the choice of the screening test.

## 5 Conclusions

In this retrospective study of average-risk individuals in a Midwest healthcare system, adherence to CRC screening increased from 35.6 % in 2015 to 45.6 % in 2018. For those aged 50–75 years at the time of the study, this increase was 47 % to 59 % respectively. Moreover, among those who were up to date with their CRC screening, the proportion of members receiving an mt-sDNA screening test after age 45 years or 50 years increased significantly from 2015 to 2018. Our results suggest that the clinical availability and growing adoption of mt-sDNA may be correlated with an increase in overall screening in this average-risk population, in parallel with a slight increase in the use of other stool-based CRC screening tests.

## Declaration of Competing Interest

The authors declare that they have no known competing financial interests or personal relationships that could have appeared to influence the work reported in this paper.

## Figures and Tables

**Table A1:** Patients not at average risk, and excluded from the current study, will have the following codes associated with their screening test or procedure:

**Code**	**Description**
ICD-9	
V12.72	Personal history of colonic polyps
V10.05	Personal history of malignant neoplasm of the large colon
V10.06	Personal history of malignant neoplasm of the rectum, rectosigmoid junction, anus
V16.0	Family history of malignant neoplasm of the gastrointestinal tract
V84.09	Genetic susceptibility to other malignant neoplasm (includes lynch syndrome)
153	Malignant neoplasm of colon
153.0	Malignant neoplasm of hepatic flexure
153.1	Malignant neoplasm of transverse colon
153.2	Malignant neoplasm of descending colon
153.3	Malignant neoplasm of sigmoid colon
153.4	Malignant neoplasm of cecum
153.5	Malignant neoplasm of appendix vermiformis
153.6	Malignant neoplasm of ascending colon
153.7	Malignant neoplasm of splenic flexure
153.8	Malignant neoplasm of other specified sites of large intestine
153.9	Malignant neoplasm of colon, unspecified
154	Malignant neoplasm of rectum, rectosigmoid junction, and anus
154.0	Malignant neoplasm of rectosigmoid junction
154.1	Malignant neoplasm of rectum
154.8	Malignant neoplasm of other sites of rectum, rectosigmoid junction, and anus
230.3	Carcinoma in situ of colon
230.4	Carcinoma in situ of rectum
211.3	Benign neoplasm of colon (includes Familial Adenomatous Polyposis)
211.4	Benign neoplasm of rectum and anal canal
569.0	Anal and rectal polyp
569.49	Other specified disorders of rectum and anus (includes fibroepitheloid polyp)
555	Regional enteritis
555.0	Crohn’s disease (inflammatory bowel disease)
555.1	Crohn’s disease (inflammatory bowel disease),
555.2	Crohn’s disease (inflammatory bowel disease)
555.9	Crohn’s disease (inflammatory bowel disease)
556	Ulcerative proctocolitis
556.0–556.9	Ulcerative Colitis
578.1	Stool contents: occult blood in stool
792.1	Stool contents: abnormal substance in stool

ICD-10	
K50.00	Crohn's disease of small intestine without complications
K50.011	Crohn’s disease of small intestine with rectal bleeding
K50.012	Crohn's disease of small intestine with intestinal obstruction
K50.013	Crohn's disease of small intestine with fistula
K50.014	Crohn's disease of small intestine with abscess
K50.018	Crohn's disease of small intestine with other complication
K50.019	Crohn's disease of small intestine with unspecified complications
K50.811	Crohn's disease of both small and large intestine with rectal bleeding
K50.812	Crohn's disease of both small and large intestine with intestinal obstruction
K50.813	Crohn's disease of both small and large intestine with fistula
K50.814	Crohn's disease of both small and large intestine with abscess
K50.818	Crohn's disease of both small and large intestine with other complication
K50.819	Crohn's disease of both small and large intestine with unspecified complications
K51.011	Ulcerative (chronic) pancolitis with rectal bleeding
K51.012	Ulcerative (chronic) pancolitis with intestinal obstruction
K51.013	Ulcerative (chronic) pancolitis with fistula
K51.014	Ulcerative (chronic) pancolitis with abscess
K51.018	Ulcerative (chronic) pancolitis with other complication
K51.019	Ulcerative (chronic) pancolitis with unspecified complications
K51.211	Ulcerative (chronic) proctitis with rectal bleeding
K51.212	Ulcerative (chronic) proctitis with intestinal obstruction
K51.213	Ulcerative (chronic) proctitis with fistula
K51.214	Ulcerative (chronic) proctitis with abscess
K51.218	Ulcerative (chronic) proctitis with other complication
K51.219	Ulcerative (chronic) proctitis with unspecified complications
K51.40	Inflammatory polyps of colon without complications
K51.411	Inflammatory polyps of colon with rectal bleeding
K51.412	Inflammatory polyps of colon with intestinal obstruction
K51.413	Inflammatory polyps of colon with fistula
K51.414	Inflammatory polyps of colon with abscess
K51.418	Inflammatory polyps of colon with other complication
K51.419	Inflammatory polyps of colon with unspecified complications
K51.50	Left sided colitis without complications
K51.511	Left sided colitis with rectal bleeding
K51.512	Left sided colitis with intestinal obstruction
K51.513	Left sided colitis with fistula
K51.514	Left sided colitis with abscess
K51.518	Left sided colitis with other complication
K51.519	Left sided colitis with unspecified complications
K51.80	Other ulcerative colitis without complications
K51.811	Other ulcerative colitis with rectal bleeding
K51.812	Other ulcerative colitis with intestinal obstruction
K51.813	Other ulcerative colitis with fistula
K51.814	Other ulcerative colitis with abscess
K51.818	Other ulcerative colitis with other complication
K51.819	Other ulcerative colitis with unspecified complications
K52.8	Other specified noninfective gastroenteritis and colitis
K62.1	Rectal polyp
K62.5	Hematochezia
K50.8	Crohn’s disease of small and large intestine without complication
K50.10	Crohn’s disease of large intestine without complication
K50.11	Crohn’s disease of large intestine
K50.111	Crohn’s disease of large intestine with rectal bleeding
K50.112	Crohn’s disease of large intestine with intestinal obstruction
K50.113	Crohn’s disease of large intestine with fistula
K50.114	Crohn’s disease of large intestine with abscess
K50.118	Crohn’s disease of large intestine with other complication
K50.119	Crohn’s disease of large intestine with unspecified complications
K50.80	Crohn’s disease of both small and large intestine without complications
K50.90	Crohn’s disease, unspecified, without complications
K50.911	Crohn’s disease, unspecified, with rectal bleeding
K50.912	Crohn’s disease, unspecified, with intestinal obstruction
K50.913	Crohn’s disease, unspecified, with fistula
K50.914	Crohn’s disease, unspecified, with abscess
K50.918	Crohn’s disease, unspecified, with other complication
K50.919	Crohn’s disease, unspecified, with unspecified complications
K51.00	Ulcerative (chronic) pancolitis without complications
K51.90	Ulcerative colitis, unspecified, without complication
K51.911	Ulcerative colitis, unspecified with rectal bleeding
K51.912	Ulcerative colitis, unspecified with intestinal obstruction
K51.913	Ulcerative colitis, unspecified with fistula
K51.914	Ulcerative colitis, unspecified with abscess
K51.918	Ulcerative colitis, unspecified with other complication
K51.919	Ulcerative colitis, unspecified with unspecified complications
K51.20	Ulcerative (chronic) proctitis
K51.3	Ulcerative (chronic) rectosigmoiditis
K51.30	Ulcerative (chronic) rectosigmoiditis without complications
K51.311	Ulcerative (chronic) rectosigmoiditis with rectal bleeding
K51.312	Ulcerative (chronic) rectosigmoiditis with intestinal obstruction
K51.313	Ulcerative (chronic) rectosigmoiditis with fistula
K51.314	Ulcerative (chronic) rectosigmoiditis with abscess
K51.318	Ulcerative (chronic) rectosigmoiditis with other complication
K51.319	Ulcerative (chronic) rectosigmoiditis with unspecified complications
K92.1	Melena
R19.5	Other fecal abnormalities (FOBT/FIT positives, occult blood)
Z86.01	Personal history of adenoma/colonic polyps
Z86.010	Personal history of colonic polyps
Z85.09	Personal history of malignant neoplasm digestive tract
Z15.09	Genetic susceptibility to other malignant neoplasm
C18.0	Malignant neoplasm of cecum
C18.1	Malignant neoplasm of appendix
C18.3	Malignant neoplasm of hepatic flexure
C18.5	Malignant neoplasm of splenic flexure
C18.7	Malignant neoplasm of sigmoid colon
C18.2	Malignant neoplasm of ascending colon
C18.4	Malignant neoplasm of transverse colon
C18.6	Malignant neoplasm of descending colon
C18.8	Malignant neoplasm of overlapping sites of colon
C18.9	Malignant neoplasm of the colon, unspecified
C19	Malignant neoplasm of rectosigmoid junction
C20	Malignant neoplasm of rectum
C21.2	Malignant neoplasm of cloacogenic zone
C21.8	Malignant neoplasm of overlapping sites of rectum, anus and anal canal
D01.0	Carcinoma in situ of colon
D01.1	Carcinoma in situ of rectosigmoid junction
D01.2	Carcinoma in situ of rectum
D01.40	Carcinoma in situ of unspecified part of intestine
D01.49	Carcinoma in situ of other parts of intestine
D12.6	Adenomatous polyp of colon (benign neoplasm of colon, unspecified) includes familial adenomatous polyposis/hereditary
D12.0	Benign neoplasm of cecum (includes familial polyposis)
D12.1	Benign neoplasm of appendix (includes familial polyposis)
D12.4	Benign neoplasm of ascending colon
D12.3	Benign neoplasm of transverse colon
D12.2	Benign neoplasm of descending colon
D12.5	Benign neoplasm of sigmoid colon
D12.7	Benign neoplasm of rectosigmoid junction (includes familial polyposis)
D12.8	Benign neoplasm of rectu 1962488700 *m*

**Table A2:** Demographic characteristics (aged 40 + years).

	**01/01/2015–12/31/2015**		**01/01/2016–12/31/2016**		**01/01/2017–12/31/2017**		**01/01/2018–12/31/2018**	
	N	%	N	%	N	%	N	%
**Total**	127,348	100 %	130,341	100 %	130,013	100 %	128,026	100 %
**Age as of Dec 31st**								
40–44	8,655	6.80 %	8,936	6.86 %	8,957	6.89 %	8,814	6.88 %
45–49	15,618	12.26 %	15,518	11.91 %	15,105	11.62 %	14,440	11.28 %
50–75	82,553	64.82 %	84,952	65.18 %	84,941	65.33 %	83,721	65.39 %
≥76	20,522	16.11 %	20,935	16.06 %	21,010	16.16 %	21,051	16.44 %
**Sex**								
Males	57,044	44.79 %	59,115	45.35 %	59,287	45.60 %	58,394	45.61 %
Females	70,304	55.21 %	71,226	54.65 %	70,726	54.40 %	69,632	54.39 %
**Race**								
American Indian /Pacific Islander	328	0.26 %	359	0.28 %	374	0.29 %	370	0.29 %
Asian	1,805	1.42 %	1,980	1.52 %	2,089	1.61 %	2,250	1.76 %
Black	15,691	12.32 %	16,408	12.59 %	16,565	12.74 %	16,052	12.54 %
White	104,761	82.26 %	106,676	81.84 %	106,133	81.63 %	104,780	81.84 %
Other	4,763	3.74 %	4,918	3.77 %	4,852	3.73 %	4,574	3.57 %
**Hispanic**								
Yes	6,777	5.32 %	7,403	5.68 %	7,864	6.05 %	8,126	6.35 %
No	120,571	94.68 %	122,938	94.32 %	122,149	93.95 %	119,900	93.65 %
**Payment type**								
Commercial	12,485	9.80 %	13,924	10.68 %	14,956	11.50 %	15,663	12.23 %
Medicare	25,389	19.94 %	25,606	19.65 %	24,933	19.18 %	23,521	18.37 %
Medicaid	1,567	1.23 %	1,755	1.35 %	1,877	1.44 %	1,766	1.38 %
Self Pay	5,282	4.15 %	5,914	4.54 %	6,130	4.71 %	5,508	4.30 %
Other	72,391	56.85 %	77,917	59.78 %	81,327	62.55 %	81,552	63.70 %
Unknown	10,234	8.04 %	5,225	4.01 %	790	0.61 %	16	0.01 %

**Table A3:** CRC Screening Test Mix – Proportion: Overall and by age groups (<50 and > 75 years).

	**01/01/2015–12/31/2015**	**01/01/2016–12/31/2016**	**01/01/2017–12/31/2017**	**01/01/2018–12/31/2018**	**Linear trend**	
					**p-value**	**direction**
**Overall (40 + )**						
High sensitivity gFOBT (gFOBT) / FIT	19.82 %	22.07 %	19.26 %	16.88 %	<0.0001	negative
mt-sDNA	3.28 %	4.90 %	6.84 %	10.99 %	<0.0001	positive
CT colonography	0.07 %	0.11 %	0.09 %	0.01 %	<0.05	negative
Screening Colonoscopy	76.71 %	72.60 %	73.43 %	71.67 %	<0.0001	negative
Sigmoidoscopy	0.11 %	0.32 %	0.38 %	0.45 %	<0.0001	positive
**Aged as of Dec 31st: 40 to 44**						
High sensitivity gFOBT (gFOBT) / FIT	29.05 %	34.55 %	23.67 %	18.50 %	<0.01	negative
mt-sDNA	0.48 %	0.00 %	0.00 %	0.00 %	NS	N/A
CT colonography	0.00 %	0.00 %	0.00 %	0.00 %	NS	N/A
Screening Colonoscopy	70.48 %	64.63 %	76.33 %	79.19 %	<0.01	positive
Sigmoidoscopy	0.00 %	0.81 %	0.00 %	2.31 %	<0.1	positive
**Aged as of Dec 31st: 45 to 49**						
High sensitivity gFOBT (gFOBT) / FIT	31.32 %	27.35 %	24.31 %	18.48 %	<0.0001	negative
mt-sDNA	0.21 %	0.00 %	0.00 %	0.27 %	NS	N/A
CT colonography	0.00 %	0.00 %	0.00 %	0.00 %	NS	N/A
Screening Colonoscopy	68.27 %	72.04 %	75.26 %	79.89 %	<0.0001	positive
Sigmoidoscopy	0.21 %	0.61 %	0.42 %	1.36 %	<0.1	positive
**Aged as of Dec 31st: ≥76**						
High sensitivity gFOBT (gFOBT) / FIT	35.25 %	36.23 %	32.54 %	30.73 %	<0.01	negative
mt-sDNA	3.10 %	3.44 %	5.05 %	7.18 %	<0.0001	positive
CT colonography	0.13 %	0.48 %	0.32 %	0.00 %	NS	N/A
Screening Colonoscopy	61.20 %	59.13 %	60.74 %	61.05 %	NS	N/A
Sigmoidoscopy	0.32 %	0.72 %	1.34 %	1.05 %	<0.01	positive

**Table A4:** CRC Screening Test Mix - Screening Incidence: Overall and by age groups (<50 and > 75 years).

	**Screening incidence for those due for CRC screening, per 1000**						**Screening incidence for all, per 1000**					
	**01/01/2015–12/31/2015**	**01/01/2016–12/31/2016**	**01/01/2017–12/31/2017**	**01/01/2018–12/31/2018**	**Linear trend**		**01/01/2015–12/31/2015**	**01/01/2016–12/31/2016**	**01/01/2017–12/31/2017**	**01/01/2018–12/31/2018**	**Linear trend**	
					**p-value**	**direction**					**p-value**	**direction**
**Overall (40 + )**												
Any screening modality	101.47	109.29	108.19	103.24	NS	N/A	95.70	107.46	107.17	99.78	NS	N/A
High sensitivity gFOBT (gFOBT)	14.37	15.97	12.51	7.04	NS	N/A	14.76	17.10	13.22	8.47	NS	N/A
FIT	3.39	5.97	7.31	10.47	<0.05	positive	4.21	6.61	7.41	8.37	<0.05	positive
Mt-sDNA (COLOGUARD)	4.16	7.11	10.53	16.98	<0.05	positive	3.14	5.26	7.33	10.97	<0.05	positive
CT colonography (CTC)	0.07	0.08	0.05	0.00	NS	N/A	0.07	0.12	0.10	0.01	NS	N/A
Screening Colonoscopy (SC)	79.42	79.99	77.62	68.55	NS	N/A	73.41	78.02	78.70	71.52	NS	N/A
Sigmoidoscopy (SIG)	0.07	0.16	0.17	0.20	<0.1	positive	0.10	0.35	0.41	0.45	<0.1	positive
**Aged as of Dec 31st:** **40 to 44**												
Any screening modality	23.76	26.39	17.98	18.80	NS	N/A	24.26	27.53	18.87	19.63	NS	N/A
High sensitivity gFOBT (gFOBT)	4.97	6.88	3.32	2.44	NS	N/A	4.97	7.16	3.35	2.50	NS	N/A
FIT	2.13	2.29	1.15	1.16	NS	N/A	2.08	2.35	1.12	1.13	NS	N/A
Mt-sDNA (COLOGUARD)	0.12	0.00	0.00	0.00	NS	N/A	0.12	0.00	0.00	0.00	NS	N/A
CT colonography (CTC)	0.00	0.00	0.00	0.00	NS	N/A	0.00	0.00	0.00	0.00	NS	N/A
Screening Colonoscopy (SC)	16.55	17.10	13.52	14.85	NS	N/A	17.10	17.79	14.40	15.54	NS	N/A
Sigmoidoscopy (SIG)	0.00	0.11	0.00	0.35	NS	N/A	0.00	0.22	0.00	0.45	NS	N/A
**Aged as of Dec 31st: 45 to 49**												
Any screening modality	29.22	29.69	28.10	21.96	NS	N/A	30.67	31.58	31.31	25.48	NS	N/A
High sensitivity gFOBT (gFOBT)	7.69	5.92	5.82	3.82	<0.05	negative	7.94	6.12	6.02	3.95	<0.05	negative
FIT	1.74	2.32	1.49	0.60	NS	N/A	1.66	2.51	1.59	0.76	NS	N/A
Mt-sDNA (COLOGUARD)	0.07	0.00	0.00	0.07	NS	N/A	0.06	0.00	0.00	0.07	NS	N/A
CT colonography (CTC)	0.00	0.00	0.00	0.00	NS	N/A	0.00	0.00	0.00	0.00	NS	N/A
Screening Colonoscopy (SC)	19.72	21.31	20.72	17.16	NS	N/A	20.94	22.75	23.57	20.36	NS	N/A
Sigmoidoscopy (SIG)	0.00	0.14	0.07	0.30	NS	N/A	0.06	0.19	0.13	0.35	NS	N/A
**Aged as of Dec 31st: ≥76**												
Any screening modality	70.22	68.42	60.97	54.45	<0.05	negative	75.48	79.25	74.44	68.17	NS	N/A
High sensitivity gFOBT (gFOBT)	18.69	15.64	14.21	11.07	<0.01	negative	19.30	17.96	15.52	12.11	<0.05	negative
FIT	6.70	9.78	7.31	9.67	NS	N/A	7.31	10.75	8.71	8.84	NS	N/A
Mt-sDNA (COLOGUARD)	2.64	3.37	4.53	6.08	<0.05	positive	2.34	2.72	3.76	4.89	<0.05	positive
CT colonography (CTC)	0.12	0.31	0.27	0.00	NS	N/A	0.10	0.38	0.24	0.00	NS	N/A
Screening Colonoscopy (SC)	41.84	38.95	34.17	27.34	<0.05	negative	46.19	46.86	45.22	41.61	NS	N/A
Sigmoidoscopy (SIG)	0.24	0.37	0.47	0.29	NS	N/A	0.24	0.57	1.00	0.71	NS	N/A

**Table A5:** CRC Screening Test Mix - Screening Rates: Overall, and by by age groups (<50 and > 75 years).

	Screening rate for those who performed a test, per 1000							Screening rate for all, per 1000				
	01/01/2015–12/31/2015	01/01/2016–12/31/2016	01/01/2017–12/31/2017	01/01/2018–12/31/2018	Linear trend		01/01/2015–12/31/2015	01/01/2016–12/31/2016	01/01/2017–12/31/2017	01/01/2018–12/31/2018	Linear trend	
					p-value	direction					p-value	direction
**Overall (40 + )**												
Any screening modality	95.70	107.46	107.17	99.78	NS	N/A	355.70	400.46	436.73	465.48	<0.01	positive
High sensitivity gFOBT (gFOBT)	14.76	17.10	13.22	8.47	NS	N/A	20.13	25.03	24.37	21.76	NS	N/A
FIT	4.21	6.61	7.41	8.37	<0.05	positive	4.24	7.69	9.83	12.26	<0.01	positive
Mt-sDNA (COLOGUARD)	3.14	5.26	7.33	10.97	<0.05	positive	3.16	7.89	14.38	22.24	<0.01	positive
CT colonography (CTC)	0.07	0.12	0.10	0.01	NS	N/A	0.13	0.23	0.30	0.27	NS	N/A
Screening Colonoscopy (SC)	73.41	78.02	78.70	71.52	NS	N/A	327.73	359.00	387.01	407.84	<0.01	positive
Sigmoidoscopy (SIG)	0.10	0.35	0.41	0.45	<0.1	positive	0.32	0.61	0.84	1.12	<0.01	positive
**Aged as of Dec 31st: 40 to 44**												
Any screening modality	24.26	27.53	18.87	19.63	NS	N/A	45.87	50.47	42.87	40.50	NS	N/A
High sensitivity gFOBT (gFOBT)	4.97	7.16	3.35	2.50	NS	N/A	5.55	7.39	4.47	3.40	NS	N/A
FIT	2.08	2.35	1.12	1.13	NS	N/A	2.08	2.35	1.23	1.13	NS	N/A
Mt-sDNA (COLOGUARD)	0.12	0.00	0.00	0.00	NS	N/A	0.12	0.00	0.00	0.00	NS	N/A
CT colonography (CTC)	0.00	0.00	0.00	0.00	NS	N/A	0.00	0.00	0.00	0.00	NS	N/A
Screening Colonoscopy (SC)	17.10	17.79	14.40	15.54	NS	N/A	38.13	40.51	37.18	35.51	NS	N/A
Sigmoidoscopy (SIG)	0.00	0.22	0.00	0.45	NS	N/A	0.00	0.22	0.00	0.45	NS	N/A
**Aged as of Dec 31st: 45 to 49**												
Any screening modality	30.67	31.58	31.31	25.48	NS	N/A	70.24	81.78	93.35	96.19	<0.05	positive
High sensitivity gFOBT (gFOBT)	7.94	6.12	6.02	3.95	<0.05	negative	8.84	7.99	8.08	6.51	<0.1	negative
FIT	1.66	2.51	1.59	0.76	NS	N/A	1.66	2.51	1.79	0.97	NS	N/A
Mt-sDNA (COLOGUARD)	0.06	0.00	0.00	0.07	NS	N/A	0.06	0.13	0.13	0.07	NS	N/A
CT colonography (CTC)	0.00	0.00	0.00	0.00	NS	N/A	0.13	0.13	0.07	0.07	NS	N/A
Screening Colonoscopy (SC)	20.94	22.75	23.57	20.36	NS	N/A	59.42	70.76	83.02	88.02	<0.05	positive
Sigmoidoscopy (SIG)	0.06	0.19	0.13	0.35	NS	N/A	0.13	0.26	0.26	0.55	<0.1	positive
**Aged as of Dec 31st: ≥76**												
Any screening modality	75.48	79.25	74.44	68.17	NS	N/A	228.97	285.93	339.55	387.11	<0.001	positive
High sensitivity gFOBT (gFOBT)	19.30	17.96	15.52	12.11	<0.05	negative	25.34	28.33	31.46	32.11	<0.05	positive
FIT	7.31	10.75	8.71	8.84	NS	N/A	7.31	12.28	12.61	14.58	<0.1	positive
Mt-sDNA (COLOGUARD)	2.34	2.72	3.76	4.89	<0.05	positive	2.34	5.21	9.76	13.59	<0.05	positive
CT colonography (CTC)	0.10	0.38	0.24	0.00	NS	N/A	0.19	0.57	0.67	0.62	NS	N/A
Screening Colonoscopy (SC)	46.19	46.86	45.22	41.61	NS	N/A	193.11	238.36	283.20	323.98	<0.001	positive
Sigmoidoscopy (SIG)	0.24	0.57	1.00	0.71	NS	N/A	0.68	1.19	1.86	2.23	<0.01	positive

## Data Availability

The data that has been used is confidential.
